# Technological Performance of Cricket Powder (*Acheta domesticus* L.) in Wheat-Based Formulations

**DOI:** 10.3390/insects13060546

**Published:** 2022-06-14

**Authors:** Andrea Bresciani, Gaetano Cardone, Costanza Jucker, Sara Savoldelli, Alessandra Marti

**Affiliations:** Department of Food, Environmental and Nutritional Sciences (DEFENS), Università degli Studi di Milano, Via G. Celoria 2, 20133 Milan, Italy; andrea.bresciani@unimi.it (A.B.); gaetano.cardone@unimi.it (G.C.); costanza.jucker@unimi.it (C.J.); sara.savoldelli@unimi.it (S.S.)

**Keywords:** entomophagy, insect powder, rheological properties, dough, bread making, fresh pasta

## Abstract

**Simple Summary:**

Considering the chemical composition of cricket powder, specific nutritional goals (such as high protein content) can be achieved by including cricket powder, even at low levels (i.e., 5%). However, when formulating new food products, it is worth assessing the functional properties of the new ingredient and/or formulation. Thus, the aim of this study was to assess the technological properties of cricket-enriched wheat flours to be used in the development of staple foods, such as bread or pasta, whose features are also discussed.

**Abstract:**

The recent socio-economic situation requires producers to change the composition of basic foods. The aim of this study was to assess the technological properties of wheat flour enriched with cricket powder (CP) (at 5%, 10%, and 20% levels) for the development of bread and pasta. The hydration (i.e., water absorption capacity, oil absorption capacity, water absorption index, water solubility index, and swelling power), foaming (i.e., foaming capacity and stability),emulsifying (emulsifying activity and emulsion stability), and rheological (during gluten aggregation, mixing, extension, and leavening) properties were investigated. Finally, bread and fresh pasta were prepared and characterized. Emulsifying activity, stability, and foaming capacity decreased in the presence of CP, whereas foaming stability and water solubility increased. The results on dough rheology highlighted the need to increase the amount of water, and to decrease the mixing and leavening time, to keep an acceptable bread volume. Indeed, 10% CP enrichment led to a product characterized by a similar volume and crumb hardness to the control (wheat flour). Despite the decrease in extensibility caused by CP, it was possible to produce fresh pasta enriched with CP, with the best cooking behavior obtained at a 5% replacement level.

## 1. Introduction

Considering the estimated population increase of more than 9 billion in 2050 and the consequent increase in food demand [1], along with the growing need for alternative protein sources, the consumption of insects may be a viable strategy to face these challenges [2]. Insects have been consumed for years, especially in American, African, and Asian countries [2]; they are eaten either as separate dish or added in formulations, for example, in the form of powder [3]. The high nutritional value of edible insects has been attracting the attention of researchers and the food industry due to their potential use in the formulation of foods with enhanced nutritional and sustainable features [4]. Indeed, insects are a good source of proteins, essential amino acids, polyunsaturated fatty acids, minerals (e.g., iron, selenium, and zinc), and vitamins (mainly those of the B-group) [5,6].

Entomophagy within Western consumers is still limited because the consumption of insects as a protein source has never played a significant role in the food culture [7], and consumers do not seem to be ready to adopt insects in the years to come [8,9]. However, Mlček et al. [10] have suggested that using whole insects or parts of them that are not directly recognizable in food products might help to increase insect acceptability and the propensity to consume them. Therefore, in trying to achieve this goal, a few studies on the enrichment of insect powder in batters [11], bread [12,13], extruded snacks [14], and pasta [15,16] have been reported.

Several hundreds of millions of people are considered to consume insects worldwide [14,17]. Among the most-consumed insects are those belonging to the following orders: Coleoptera (their consumption represents 31% of all the edible insects), Lepidoptera (18%), Hymenoptera (14%), Orthoptera (13%), Hemiptera (10%), Odonata (3%), and Diptera (2%) [18]. The commonly known house cricket (*Acheta domesticus* L., Orthoptera Gryllidae) represents one of the most promising edible species for humans among farmed insects [2,19]. Its consumption, already common in several countries of East Asia, is mainly associated with its precious nutritional value. Recently, the house cricket has been authorized as a novel food ingredient for the EU market [20]. In fact, in addition to being an excellent source of protein (up to nearly 70% of dry matter), *A. domesticus* is also rich in minerals (e.g., calcium, iron, and magnesium) (3–10%), fat, and essential fatty acids (15–40%) [21,22].

It follows that insects are considered one of the most important food innovations of the last decade [4,6], driving the research to formulate new food products with this sustainable material. However, while formulating new food products, the effect of new ingredients on the food matrix needs to be considered, to ensure the maintenance of desirable characteristics in the final product, thus assuring consumer acceptability.

Wheat-based products represent the most-consumed products in worldwide diets. Common wheat (*Triticum aestivum* L.) is used in a wide range of applications ranging from bread, biscuits, and cakes to noodles and pasta. The functionality and versatility of flour is associated with the capacity of its storage proteins—gliadins and glutenins—to form gluten, but strongly depends on the formulation; the addition of non-gluten and/or fiber-enriched ingredients might dilute gluten as well as interfere with gluten formation. Along with the growing interest in wheat-based products with a wide range of flavors and innovative raw materials, insects could fit perfectly into this context.

To the best of our knowledge, only a few authors have investigated the effects of the replacement of wheat flour with cricket powder on dough rheology and bread-making characteristics [12,13,23]. The rheological properties of wheat are considered to be of great importance for determining technological performance, and are useful tools for predicting process efficiency and product quality [24]. The rheological properties of doughs might be assessed through a wide range of approaches. Some of these approaches determine, for instance, the amount of mixing that a dough requires, or the amount of water that should be added to the flour to obtain dough of the desired consistency (e.g., the Farinograph by Brabender GmbH & Co. KG, Duisburg, Germany). Others simulate rounding and molding in the baking process and measure the dough resistance to uni-axial extension (Extensograph by Brabender GmbH & Co. KG, Duisburg, Germnay) or to three-dimensional extension (Alveograph by Chopin Technologies, Villeneuve La Garenne Cedex, France), in order to determine the dough-strength properties; this is useful for predicting bread/pasta-making quality. More recently, the GlutoPeak (Brabender GmbH & Co. KG, Duisburg, Germany) test has been proposed to assess the gluten aggregation properties of flours [25].

This study seeks to implement and increase the value of notions about the application of cricket powder in foods. It follows the need to increase the range of products enriched with cricket powder in order to increase the market and acceptability of insects in the diet in Western countries. Regarding pasta, for example, none of the few recent studies [16,26] have dealt with fresh pasta from common wheat.

Therefore, the aim of this study was to assess the functional and rheological properties of wheat flour enriched with cricket powder, in order to select the enrichment level to be used for developing stable foods such as bread and fresh pasta.

## 2. Materials and Methods

### 2.1. Materials

Cricket powder (CP) was produced starting from a colony of *Acheta domesticus* L. present at the DeFENS (University of Milan), as reported by Jucker et al. (2021) [27]. Briefly, crickets were reared inside containers (71 × 46 × 35 cm) in a climate room at 27 ± 1 °C, 60 ± 5% relative humidity, and under a 12:12 h light:dark cycle. Each container was provided with a cardboard egg box designed to hold 30 eggs (25 × 40 cm) in order to prevent cannibalism and to ensure a sufficient surface area was available for the crickets. Hen feed (Sani sapori, Petrini, Assisi (PG), Italy) was provided ad libitum as a diet for the crickets, while water was supplied through one chicken water-feeder. Crickets were collected from the different containers between the 6th and 8th week and were fasted for 24 h. After being rinsed with water in order to eliminate feces and waste, and killed by blanching (1 min at 100 °C in boiling water), they were frozen (−18 °C) [28,29]. Frozen crickets harvested from the different containers were combined together and freeze-dried (CoolSafe Basic, LaboGene, Allerød, Denmark) for 24 h. Dried samples were finally grounded into a powder using a mill (Hawos, Queen 2, Bad Homburg, Germany). The obtained meal was sieved through a 1.25 mm mesh and kept under vacuum in a refrigerator before being used for analyses (Section 2.2). The chemical composition of CP was analyzed and described in Jucker et al. [27].

A commercial refined wheat flour (protein 14 g/100 g), provided by Molino Quaglia S.p.A. (Vighizzolo D’Este, PD, Italy), was used alone or blended with CP. Specifically, three CP:wheat flour blend ratios were studied: 5:95 (5%), 10:90 (10%), and 20:80 (20%).

### 2.2. Methods

#### 2.2.1. Functional Properties

##### Hydration Properties

The hydration properties measured were: water absorption capacity (WAC), oil absorption capacity (OAC), water absorption index (WAI), water solubility index (WSI), and swelling power (SP). The method used for WAC, OAC, WAI and WSI was developed by adapting the one proposed by Nguyen et al. [30]. The method proposed by Yadav et al. [31] was adapted to measure SP.

Each sample (3 g) was dispersed in 30 mL of distilled water or sunflower oil for WAC and OAC, respectively. The suspension was mixed in a vortex for 30 s and left to rest for 10 min; the operation was repeated three times. After that, the suspension was centrifuged (30 min at 2500× *g*), and the sediment was incubated at 50 °C for 25 min in an air oven, then weighted. The difference in the sample weight before and after hydration was calculated. The results were presented as grams of water or oil absorbed per gram of the sample, for WAC and OAC, respectively. The analysis was carried out in duplicate.

WAI, WSI, and SP indicate, respectively, the absorption of water when the flour undergoes a heating treatment, the fraction of the sample solubilized in water, and the swelling power during hydrothermal treatment [32]. In a pre-weighted tube, 5 g of sample and 30 mL of distilled water were mixed and heated for 10 min at 90 °C. After cooling, the samples were centrifuged at 2500× *g* for 12 min. The supernatant was poured in pre-weighted Petri dishes and incubated in the air oven at 110 °C for 24 h. The sediment was weighted immediately, and the day after, the dry residue in the Petri dishes was measured. WAI was calculated as the ratio between the weight after hydration and the initial weight. WSI is expressed as a percentage and represents the ratio between the weight of the residual solid after drying and the initial sample weight. SP was calculated as the difference between WAI and the weight of the residual solid after drying. The analysis was carried out in triplicate.

##### Foaming and Emulsifying Properties

Foaming capacity (*FC*) and foaming stability (*FS*) were assessed as reported by Yadav et al. [31]. Briefly, *FC* and *FS* represent the ability to form a foam and the ability to maintain a stable foam in time, respectively. They were determined by shaking 0.5 g of the sample and 25 mL of distilled water in a graduated cylinder for 5 min. The volume was measured after 5 and 60 min (*V*_0_ and *V*_1_). The analysis was carried out in triplicate and the following equations were used to determine *FC* and *FS*:

$$FC=\frac{\left(V_{0}-50\right)}{50}\times 100$$

$$FS=\frac{V_{1}}{V_{0}}\times 100$$

Emulsifying activity (EA), and emulsion stability (ES) represent the ability to form an emulsion and the ability to maintain the emulsion under stressful conditions. EA and ES were calculated by adapting Yasumatsu et al. [33]. Briefly, an emulsion was obtained by homogenizing the sample (5 g), water (100 mL) and sunflower oil (100 mL) for 1 min. The emulsion was then divided into four aliquots of 50 mL each, and placed in graduated tubes. To determine EA, two tubes were directly centrifuged (1300× *g* for 5 min), and to determine ES, the other two tubes were centrifuged after 30 min of incubation at 80 °C in the air oven. After centrifugation, at least three layers were visible, of which the central one represented the emulsion. The analysis was carried out in triplicate. The volume of the emulsion layer was registered.

EA and ES were determined as the ratio between the volume of the emulsion layer and the total volume, expressed as a percentage and adjusted by the initial sample weight. EA and ES were determined at room temperature and at 80 °C, respectively.

#### 2.2.2. Rheological Properties

##### Gluten Aggregation Properties

The gluten aggregation kinetics of samples were investigated by means of the GlutoPeak (Brabender GmbH & Co. KG, Duisburg, Germany) device. The method reported by Suárez-Estrella et al. [34] was adopted using 9 g of sample in 10 mL of water. The main indices considered were: (i) maximum torque (GPU—GlutoPeak Units), corresponding to the peak occurring as gluten aggregates; (ii) peak maximum time (s), which is the time required to achieve the maximum torque; and (iii) total energy (cm^2^), corresponding to the area under the curve from the beginning of the test up to 15 s after maximum torque, indicating the total energy required for gluten aggregation. The analysis was carried out in duplicate.

##### Dough Mixing Properties

The mixing properties of doughs were studied by means of the Farinograph-E (Brabender GmbH & Co. KG, Duisburg, Germany) device equipped with a 50 g mixing bowl, following the ICC 115/1 official method [35]. The following indices were considered: (i) water absorption (%), corresponding to the amount of water added to achieve the optimal consistency (500 Farinograph units; FU); (ii) dough development time (min), expressed as the time required to reach maximum consistency; (iii) stability (min), defined as the time from when the top of the curve reaches 500 FU to when it leaves it; and (iv) degree of softening, corresponding to the difference between the 500 FU line and the dough consistency 12 min after dough development. The analysis was carried out in duplicate.

##### Dough Extension Properties

The uni-axial and three-dimensional extension properties were investigated using the Extensograph (Brabender GmbH & Co. KG, Duisburg, Germany) and the Alveograph (Chopin Technologies, Villeneuve La Garenne Cedex, France) devices, following AACC standard methods 54–10.01 and 54–30.01 [36], respectively. As regards dough extensibility evaluated by means of the Extensograph, it was recorded at three different rest times (45, 90, and 135 min), and the following parameters were considered: (i) resistance to extension (EU—Extensograph units), which corresponds to the resistance after 5 cm from the beginning of the test and is related to elastic properties; (ii) extensibility (mm), related to the length of the curve; (iii) ratio between resistance to extension and extensibility; and (iv) energy (cm^2^), corresponding to the area under the curve. As regards the three-dimensional extension properties evaluated by the Alveograph, the main indices considered were: (i) tenacity (mmH_2_O, P), corresponding to the maximum pressure on deformation; (ii) extensibility (mm, L), corresponding to the length of the curve; (iii) P/L ratio; and (iv) force (10^−4^ J, W), corresponding to the area under the curve. The analyses were carried out in duplicate.

#### 2.2.3. Bread Making and Bread Characterization

Doughs were prepared using wheat flour alone, 10:90, and 20:80 blends, applying a straight-dough method. Specifically, flour samples were mixed with fresh yeast (2% flour) and salt (1.5% flour). The amount of water and the mixing time applied varied for each formulation, according to the farinographic indices (Supplementary Table S1). Three dough sub-samples of 250 g each were shaped into a cylindrical form, then put into bread molds (length: 12.5 cm; width: 6 cm; height: 5 cm) and left to leaven according to the leavening time determined through the Rheofermentometer test, at 30 °C (Supplementary Table S1). At the end of the proofing phase, the leavened dough was baked (SelfCooking Center^®^, Rational International AG, Landsberg am Lech, Germany) at 220 °C for 25 min. For each sample, two baking tests were performed for each sample and three loaves were obtained from each baking test.

Dough-leavening properties were investigated by means of the Rheofermentometer device (Chopin Technologies, Villeneuve La Garenne Cedex, France), in terms of dough development and carbon dioxide (CO_2_) production and retention; the dough was tested for 360 min at 30 °C, on an aliquot of 315 g of the dough prepared under the conditions given in the previous paragraph. The analysis was carried out in duplicate.

The bread specific-volume was evaluated through the ratio between volume [36] and mass of bread (mL/g). Crumb moisture was determined by means of a moisture analyzer (MA 210.R, Wagi Elektroniczne, Poland) on 3 g of crumb at 130 °C, until the weight did not change by 1 mg in 10 s. Crumb firmness was assessed by means of a texture analyzer (TA.XT-plus, Stable Micro Systems Ltd., Godalming, UK), equipped with a 100 N load cell and a 36 mm probe (Radiused Cylinder Probe—P36R). The AACC 74–09.01 [36] method was used, with a test speed of 100 mm/min. Crumb firmness was tested at 2, 24, and 48 h after baking. In particular, specific volume was replicated six times and crumb moisture was replicated three times, while crumb firmness was measured on the three central slices of each bread obtained from each baking trial.

#### 2.2.4. Fresh Pasta Preparation and Characterization

Water was added to the samples (wheat flour alone, 5:95, and 10:90 blends) in order to obtain dough with 40 g/100 g final moisture. Samples were kneaded for 10 min using an automatic spiral kneader (KitchenAid, model 5KSM150, Benton Harbor, MI, USA). The resulting dough was covered with plastic film for a rest period of 15 min. The thickness of the pasta sheet was gradually reduced using a pasta roller (KitchenAid, model KSMPSA) through three consecutive steps up to 2 mm. Pasta dough was shaped into “tagliatelle” (length: 200 mm; width: 13.5 mm; thickness: 2.0 mm). Pasta was cooked in distilled water (pasta:water ratio = 1:10) at the optimum cooking time (90 s). After cooking, pasta was drained for 30 s, and the water absorption was determined gravimetrically. Then, the cooking water was collected, brought back to the initial volume, and an aliquot (40 mL) was dried to a constant weight at 120 °C for 15 h, to evaluate the cooking losses (g of solid loss/100 g of pasta). Finally, the firmness of the cooked pasta was evaluated on 5 “tagliatelle” by means of the Texture Analyzer TA.XT-plus (Stable Micro Systems Ltd., Godalming, UK), equipped with a light knife blade (A/LKB-F) and a 100 N load cell (crosshead speed of 10.0 mm/min), according to the AACC 66–52.01 [36] with modifications. Specifically, pasta firmness was directly assessed after a 30-s drain phase. For each sample, two pasta-making trials were carried out. Water absorption, cooking loss, and pasta firmness were analyzed in triplicate.

#### 2.2.5. Statistics

The results are expressed as mean ± standard deviation. To determine differences between wheat and CP-blends and related bread and pasta samples, the following dependent variables were compared using analysis of variance (one-way ANOVA; α = 0.05): WAC, OAC, WAI, WSI, SP, FC, FS, EA, ES, bread volume and specific volume, crumb moisture and firmness, water absorption, cooking loss, and firmness of fresh pasta. Prior to analyses, all data were examined using Levene’s test for homogeneity of variance. All statistical tests were performed using Statgraphics Plus 5.1 (StatPoint Inc., Warrenton, VA, USA) using the samples as factors. The significant differences (*p* ≤ 0.05) were determined using Tukey’s HSD test.

## 3. Results and Discussion

### 3.1. Functional Properties

When formulating new food products, it is worth assessing the functional properties of the blends. To the best of our knowledge, for the first time, the functional properties of CP–wheat blends are addressed herein. This approach was chosen to provide a direct insight into the behavior of blends in the food system. Despite the behavior of the single components, the interactions among them might affect the functional properties of the blends.

#### 3.1.1. Hydration Properties

The chemical composition of CP did not significantly affect the functional properties of the blends (Table 1), at least at the level used in the present study. Despite the high amounts of proteins, chitosan, and fat in CP, neither the WAC nor the OAC of the CP blends were different from the values found for wheat flour. Previous studies highlighted that either cricket/insect powder or proteins isolated from it can be used to increase both WHC and OHC. Ndiritu et al. [37] found protein extracts to range from 2.0 to 2.7 g/g for WHC, and 3.37–3.53 g/g for OHC, depending on the extraction method; meanwhile, Zielińska et al. [38] reported cricket-protein extracts to have higher WHC and OHC than their respective powders.

WAI, WSI, and SP were investigated to provide information about blend behavior during processing (i.e., heating). WAI and SP are strongly affected by starch granules, which absorb water and gelatinize upon heating in excess water. Specifically, WAI measures the amount of water absorbed, whereas SP measures the water entrapped within the starchy gel network during heating and stirring in excess water [39]. CP did not contribute to such phenomena, since it does not contain starch; thus, no significant differences in WAI and SP were observed among the samples. On the other hand, the amounts of sugars and soluble proteins might account for the increase in the amount of material solubilized in the system. solubility of biomolecules (starches, water-soluble fibers, proteins and/or sugars, etc.), increased in both the 10% and 20% CP blends.

#### 3.1.2. Foaming and Emulsifying Properties

As far as FC and emulsifying properties (i.e., EA and ES) are concerned, CP negatively affects these indices, regardless of the percentage in which it is added (Table 1), whereas an increase in FS was measured in the blends. EA reflects the ability and capacity of proteins to aid in the formation of emulsions, and is related to protein’s ability to absorb the interfacial area of oil and water in an emulsion. ES normally reflects the protein’s ability to strengthen an emulsion’s resistance under stress conditions and changes. This index is, therefore, related to the consistency of the interfacial area over a defined time period [40]. It has been shown that chitin does not produce emulsions [41].

FC and FS generally depend on the interfacial film, formed by proteins, which keeps air bubbles in suspension and slows down the rate of coalescence. Foaming properties are dependent on the proteins, as well as on other components such as carbohydrates present in the flour [42]. Specifically, globular proteins and sugars were found to have a reduced ability to unfold at the air–water interface, which limits the capacity to encapsulate air bubbles [43]. Cricket proteins were shown to have poor or no FC [37,44]. Although CP significantly decreased the FC of wheat, the FS improved (Table 1). The data suggested that protein–protein interactions in the system were reduced by the presence of CP; however, such interactions resulted in the formation of interfacial membranes able to stabilize the foams. In food technology, the capacity of an ingredient to form/stabilize foams is exploited to improve the texture, consistency, and appearance.

### 3.2. Rheological Properties

#### 3.2.1. Gluten Aggregation Properties

**Figure 1:**
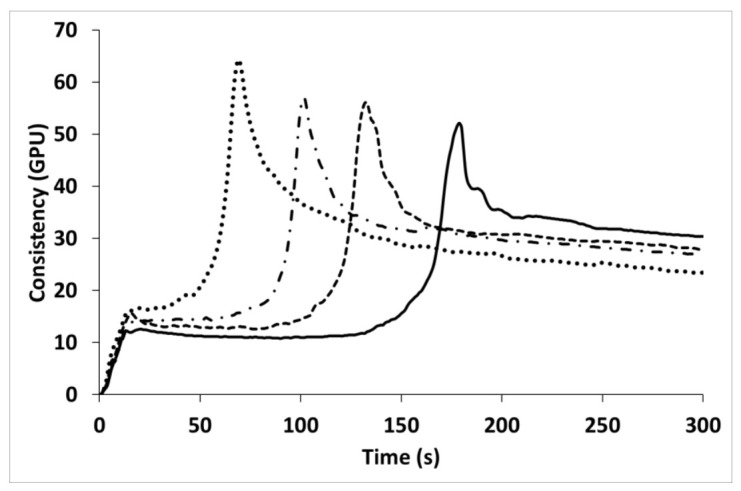
Gluten aggregation properties of wheat (solid line), and with increasing replacement level of cricket powder (5%: dashed line; 10%: dash–dotted line; 20%: dotted line). GPU—GlutoPeak Units.

Wheat flour was characterized by taking a long time to achieve gluten aggregation and high consistency; this is typical of the strong wheat flour used in bread making [45]. CP increased the maximum consistency and decreased both the peak maximum time and total energy (Figure 1 and Supplementary Table S1), suggesting gluten weakening. Gluten dilution might account for the faster aggregation (i.e., the peak maximum time decreased upon CP addition); meanwhile, the increase in protein content was responsible for the increase in peak consistency, and chitin was responsible for the decrease in total energy (Figure 1 and Supplementary Table S1). Indeed, protein content amounted to 71.8% of the dry basis, and chitin to 7.76% of the dry basis in CP (data not shown). Since no studies have previously evaluated the influence of cricket powder on the gluten aggregation kinetics of wheat, it is difficult to compare our results with the literature. However, a similar effect on gluten aggregation properties was found when bran (which is rich in fiber and soluble proteins) was added to wheat [45].

#### 3.2.2. Dough Mixing Properties

In a dough system, 5% CP did not affect water absorption, i.e., the amount of water required to form dough with optimal consistency (i.e., 500 FU). Instead, this index increased in 10 and 20% CP blends (Table 1). Similar findings were discovered by Cappelli et al. [13], who reported that water absorption increased as the amount of CP increased from 5 to 15%. In contrast, Osimani et al. [23] showed that CP up to 30% did not affect water absorption, while González et al. [12] highlighted a decrease in this index, even at a low level (i.e., 5%). Differences among the studies might be related to differences in cricket composition, especially the protein fractions [13,23].

**Figure 2:**
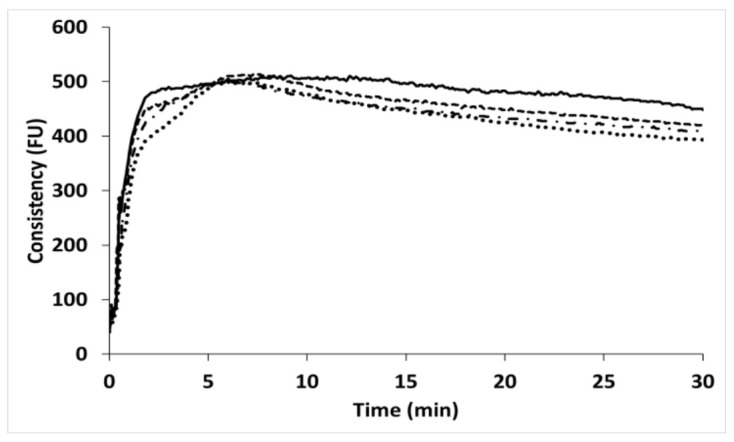
Mixing properties of wheat (solid line), and increasing replacement level of cricket powder (5%: dash line; 10%: dash–dotted line; 20%: dotted line). FU—Farinograph Units.

Dough development time decreased in CP blends, probably due to gluten dilution; however, it did not change from the 10 to 20% replacement levels (Figure 2). Osimani et al. [23] and Cappelli et al. [13] only reported a decrease in this index at the 15 and 30% replacement levels, respectively. As regards stability time and degree of softening, the indices decreased and increased, respectively; this already occurred in the 5% CP blend, with no further decrease at higher levels. In contrast, an increase in stability and a decrease in the degree of softening were measured when CP was used up to 15% [13,23]. The authors reported that changes in the farinographic indices were related to interactions between gluten and cricket proteins, and depended on their ratio [12,13,23].

#### 3.2.3. Dough Extension Properties

**Figure 3:**
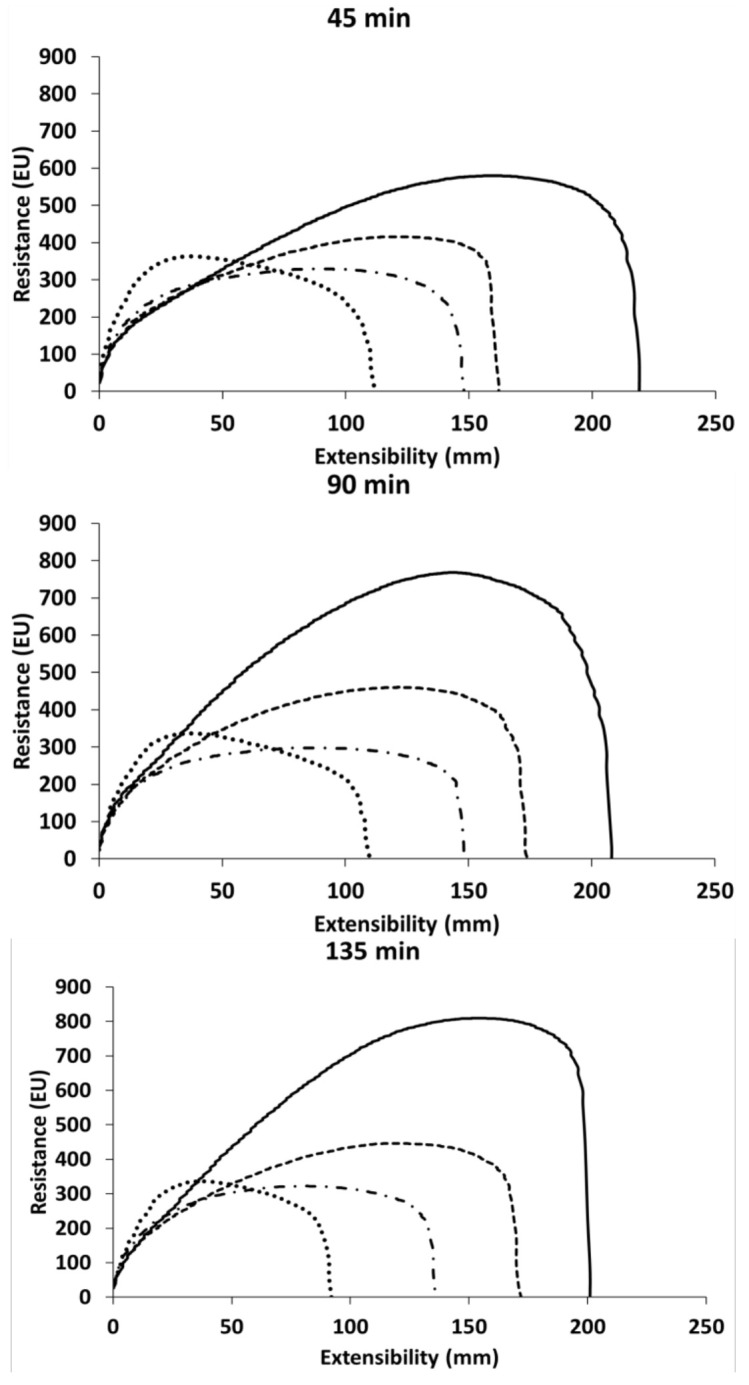
Extensional properties of wheat (solid line), and with increasing replacement level of cricket powder (5%: dash line; 10%: dash–dotted line; 20%: dotted line), after 45 min, 90 min, and 135 min of resting time. EU—Extensograph Units.

Replacing wheat flour with CP decreased dough extensibility (Figure 3 and Supplementary Table S1). In addition, no differences were observed with regard to the resting time (45, 90, and 135 min). Doughs containing 5% and 10% CP were less resistant to extension than wheat dough; no difference between wheat dough and the 20% CP blend was found. As regards the ratio number (i.e., resistance to extension and extensibility ratio), it slightly increased due to the addition of CP (Supplementary Table S1). Finally, the energy required for deformation decreased in 5% and 10% CP blends, with no further decrease at a higher CP level (i.e., 20%). These findings were similar to those reported by Osimani et al. [23] using 30% enrichment levels.

Regarding the three-dimensional extension properties of doughs, the test was performed up to the 10% replacement level, since 20% CP resulted in a dough not able to withstand the overpressure needed to blow the dough. Adding CP increased the tenacity and decreased the extensibility indices, indicating an increase in dough stiffness. This finding was also confirmed by the increase in the P/L index (i.e., tenacity and extensibility ratio). Our results are consistent with previous studies [13,23].

A significant correlation between three-dimensional and uni-axial extension indices was shown [46]. The high protein and chitin content in CP blends may explain the increase in tenacity and the decrease in extensibility, confirming previous studies [13,23]. Ghoshal and Mehta [47] pointed out that with an increase in the percentage of chitosan (a derivate of chitin) from 0.5% to 1%, the dough extensibility increased, then decreased with further increases in chitosan percentages of 1.5–2%. Moreover, the force required for deformation (W) decreased as CP increased. This behavior might be due to the gluten dilution because of wheat replacement with a high gluten-non-containing raw material and the resulting difficulty in forming a good gluten network [48]. Similar results were reported by Osimani et al. [23], wherein CP was used up to 30%. In contrast, Cappelli et al. [13] reported a slight increase in this index when wheat flour was replaced with up to 15% CP.

Overall, the rheological properties showed that in a dough system, CP might be included by up to 20%, with no further worsening compared to the 5% level (except for the absorption of more water). For these reasons, blends with 10% and 20% CP were used for bread making. However, since it was not possible to perform the alveograph test on the 20% blend, this was not used for pasta making.

### 3.3. Bread-Making Performance

#### 3.3.1. Dough-Leavening Properties

**Figure 4:**
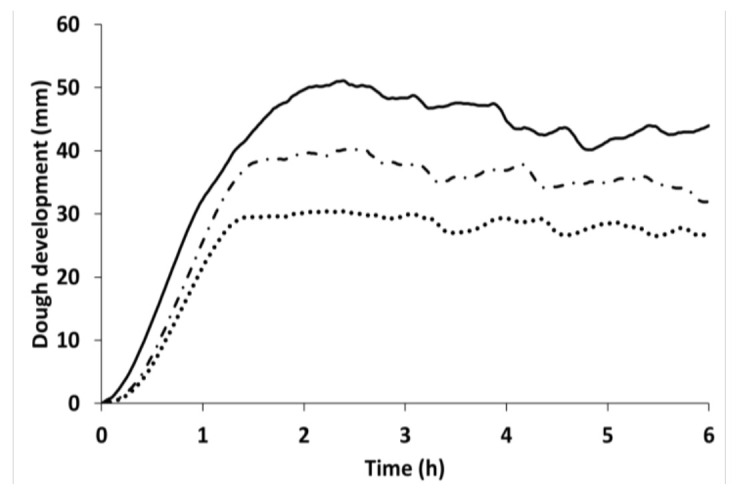
Dough-leavening properties of wheat (solid line), and with increasing replacement level of cricket powder (10%: dash–dotted line; 20%: dotted line).

Bread was prepared using CP at 10% and 20% levels. CP caused a worsening of the dough-leavening properties, in terms of both dough development and gas production (Figure 4 and Supplementary Table S1). This can be observed from the maximum dough height reached both during the test and at the end of the test; this decreased in CP-containing doughs, with the most intense worsening observed at the highest replacement level (Figure 4 and Supplementary Table S1). Moreover, CP-based doughs required longer leavening time to reach the maximum dough height, compared to wheat dough. Finally, gas production decreased only when CP was present at the highest level; meanwhile, the retained CO_2_ decreased starting from the 10% replacement level, with no further decrease up to 20% (Supplementary Table S1). Moreover, CO_2_ release decreased and retention increased in relation to the replacement level. The decrease in the released CO_2_ index, observed in CP samples, could be explained by the lower CO_2_ production.

#### 3.3.2. Bread Characteristics

**Figure 5:**
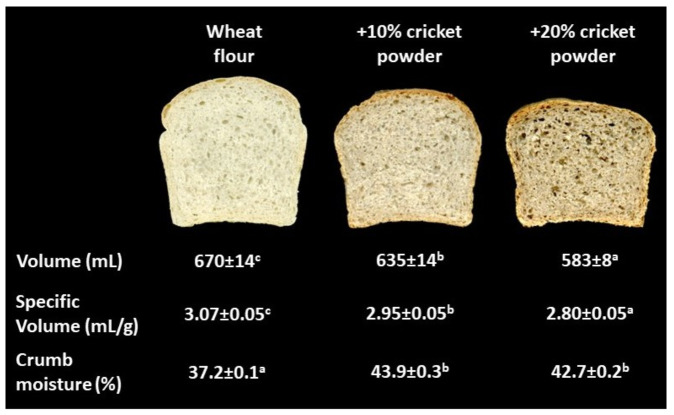
Pictures of the crumb, and quality indices (volume, specific volume, and crumb moisture) of bread prepared from wheat flour alone, and with increasing replacement level of cricket powder (10% and 20%). Data are expressed as mean ± standard deviation. Different letters in the same row correspond to significant differences (one-way ANOVA, Tukey’s HSD test, *p* ≤ 0.05).

CP decreased both the loaf volume and specific volume (Figure 5), especially at the 20% replacement level. In fact, the 20% CP bread was characterized by the lowest volume and the lowest specific volume; this was likely due to a decrease in dough extensibility and the weakening of the gluten network, which inevitably decreased the gas-retention capacity [12].

**Figure 6:**
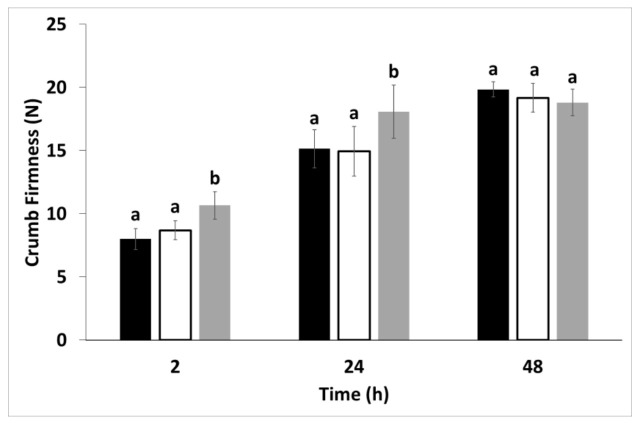
Crumb firmness of bread prepared from wheat flour alone (black), and with increasing replacement level of cricket powder (10%: white; 20%: grey), after 2, 24 and 48 h of baking. Data are expressed as mean ± standard deviation. Different letters at the same storage time indicate significant differences (one-way ANOVA, Tukey’s HSD test, *p* ≤ 0.05).

At the lowest replacement level, CP affect crumb softness neither in fresh bread nor during storage (Figure 6); on the contrary, crumb firmness increased in bread containing 20% CP and stored for one day. After 48 h of storage, no difference in crumb firmness was observed among samples. The increase in crumb firmness might be justified by the decrease in bread specific-volume (Figure 5). This negative correlation between specific volume and crumb firmness due to the compression of gas cells has been previously reported [49,50]. These results agree with those shown by de Oliveira et al. [51], who found a decrease in volume and crumb firmness in accordance with the increase in the amount of insect powder (*Nauphoeta cinerea*) added. Conversely, Osimani et al. [23] did not observe a difference in terms of specific volume between bread from wheat flour and 10% CP enrichment level, and reported an increase in crumb firmness from the 10% replacement level. In studying the effect of chitosan, Ghoshal and Mehta [47] found that a low level of chitosan (0.5%) did not affect bread volume; however, when 1 to 2% chitosan was added, the difference in volume was quite significant. An increase in firmness as the percentage of chitosan (0.5–2%) was also shown.

### 3.4. Pasta Properties

Fresh pasta was prepared up to a maximum replacement level of 10%, as larger amounts of CP led to excessive quality-worsening in terms of dough tenacity and strength (data not shown). Enriching pasta with CP increased both water absorption and cooking loss at the 5 and 10% replacement levels, respectively (Table 2). These parameters could be caused by the addition of cricket powder, which can interfere with the proteins of the gluten network, making starch more capable of swelling and, therefore, gelatinizing and absorbing water. Pasta characterized by weak gluten generally loses solid material in the cooking water. Pasta firmness was not affected by the addition of 5% CP, but it decreased at the 10% replacement level. Since, to the best of our knowledge, no study has previously evaluated the effect of cricket powder on fresh common wheat pasta, it is difficult to compare our results with the literature. However, the enriched pasta produced under these conditions was characterized by acceptable technological and structural characteristics.

## 4. Conclusions

The aim of this study was to assess the technological properties of CP-enriched wheat flours to be used in the development of staple foods, such as bread or pasta. Considering the chemical composition of the CP, specific nutritional goals (such as high protein content and fiber) can be achieved, even at low CP levels (e.g., 5%). However, when reformulating food products, it is worth assessing the functional and rheological properties of the blends. Considering the functional properties, EA, ES, and FC decreased, while FS and WSI increased, upon CP addition. The gluten-weakening in the CP blends, highlighted by rheological tests, suggest a need to increase the amount of water, and to decrease both the mixing and leavening time to avoid excessive dough-collapse during baking. Accordingly, bread with an acceptable volume and a crumb hardness similar to that of the control was made with 10% CP. Despite the decrease in extensibility, it was possible to make fresh pasta enriched with CP, exploiting the high tenacity and resistance to extension shown by CP blends. The best cooking behavior was obtained at the 5% replacement level.

Further studies might address the effect of CP on the cooking behavior of dry pasta, the upscaling of the processes, and the product’s overall acceptability.

## Figures and Tables

**Table 1:** Functional properties of wheat-cricket powder blends.

			Wheat Flour	5% Cricket Powder	10% Cricket Powder	20% Cricket Powder
Absorption properties (25 °C)	Water (WAC)	g/mL	1.69 ± 0.03 ^a^	1.69 ± 0.06 ^a^	1.73 ± 0.05 ^a^	1.75 ± 0.02 ^a^
	Oil (OAC)	g/mL	1.71 ± 0.01 ^a^	1.72 ± 0.02 ^a^	1.74 ± 0.02 ^a^	1.81 ± 0.01 ^b^
Absorption properties (90 °C)	Water absorption index (WAI)	g/g	3.73 ± 0.12 ^a^	3.81 ± 0.11 ^a^	3.86 ± 0.04 ^a^	3.85 ± 0.14 ^a^
	Water solubility index (WSI)	g/g	1.55 ± 0.16 ^a^	1.64 ± 0.38 ^a^	1.83 ± 0.09 ^ab^	2.30 ± 0.01 ^b^
Swelling power	Swelling power (SP)	g/g	3.65 ± 0.11 ^a^	3.73 ± 0.09 ^a^	3.73 ± 0.10 ^a^	3.74 ± 0.14 ^a^
Foaming capacity	Foaming capacity (FC)	%	44.00 ± 5.66 ^b^	4.00 ± 2.83 ^a^	5.00 ± 1.41 ^a^	6.00 ± 2.83 ^a^
	Foaming stability (FS)	%	90.27 ± 0.15 ^a^	98.11 ± 7.12 ^a^	98.09 ± 0.01 ^b^	98.15 ± 6.86 ^b^
Emulsifying capacity	Emulsifying activity (EA)	%	12.40 ± 0.32 ^b^	4.00 ± 0.01 ^a^	4.40 ± 0.01 ^a^	4.08 ± 0.01 ^a^
	Emulsion stability (ES)	%	11.20 ± 0.01 ^b^	3.92 ± 0.01 ^a^	3.92 ± 0.01 ^a^	2.40 ± 1.28 ^a^

Data are expressed as mean ± standard deviation. Different letters in the same row correspond to significant differences (one-way ANOVA, Tukey’s HSD test, *p* ≤ 0.05).

**Table 2:** Water absorption, cooking loss, and firmness of “tagliatelle” prepared from wheat flour alone, and with increasing replacement level of cricket powder (5% and 10% CP).

		Wheat Flour	5% CP	10% CP
Water absorption	(%)	27 ± 2 ^a^	32 ± 2 ^b^	35 ± 3 ^b^
Cooking loss	(%)	1.2 ± 0.1 ^a^	1.2 ± 0.1 ^a^	1.4 ± 0.1 ^b^
Firmness	(N)	12 ± 1 ^b^	11 ± 1 ^b^	6 ± 1 ^a^

Data are expressed as mean ± standard deviation. Different letters in the same row correspond to significant differences (one-way ANOVA, Tukey’s HSD test, *p* ≤ 0.05).

## Data Availability

Data are contained within the article or Supplementary Material.

## Supplementary Materials

The following supporting information can be downloaded at: https://www.mdpi.com/article/10.3390/insects13060546/s1, Table S1: Gluten aggregation, mixing, and extensional and leavening properties of wheat, and with increasing replacement level of cricket powder (5%, 10%, and 20% CP).
