# Supplementary material for: Technological Performance of Cricket Powder (Acheta domesticus L.) in Wheat-Based Formulations
Source: Insects. 2022 Jun 14;13(6):546. doi: 10.3390/insects13060546 (PMC9224782; doi:10.3390/insects13060546)
Supplement: Supplementary file 1 [file insects-13-00546-s001.zip › insects-1766908-supplementary.pdf]

Supplementary Table S1: Gluten aggregation, mixing, extensional, and leavening properties of wheat and with increasing replacement level of cricket powder (5%, 10%, and 20% CP).

			**Wheat flour**	**5% CP**	**10% CP**	**20% CP**
GLUTEN AGGREGATION PROPERTIES (GlutoPeak Test)	Peak maximum time	(s)	186±10[d]	131±4[c]	99±6[b]	71±2[a]
	Maximum torque	(GPU)	52.3±0.6[a]	56±0.2[b]	57.0±1.0[b]	63.5±0.7[c]
	Total Energy	(cm^2)	3402±205[c]	2882±16[b]	2454±106[a]	2294±31[a]
MIXING PROPERTIES (Farinograph Test)	Water absorption	(%)	63.1±0.3[a]	63.5±0.1[a]	65.0±0.1[b]	69.4±0.1[c]
	Development time	(min)	9.5±0.4[c]	7.6±0.4[b]	5.9±0.1[a]	6.2±0.2[a]
	Stability	(min)	20.8±1.3[b]	9.1±1.5[a]	7.2±0.6[a]	6.3±0.2[a]
	Degree of softening	(FU)	27.5±6.4[a]	59.0±7.1[b]	59.0±2.8[b]	70.0±4.2[b]
EXTENSIONAL PROPERTIES (Extensograph Test)	Extensibility (E)_45	(mm)	210±13[c]	169±9[b]	147±1[b]	111±1[a]
	Resistance to extension (R)_45	(EU)	328±1[ab]	323±16[ab]	306±2[a]	356±1[b]
	R/E ratio45		1.6±0.1[a]	1.9±0.1[b]	2.1±0.1[b]	3.2±0.1[c]
	Energy_45 min	(cm^2)	161±11[c]	103±11[b]	73±1[ab]	58±1[a]
	Extensibility (E)_90	(mm)	202±9[d]	172±3[c]	144±6[b]	107±4[a]
	Resistance to extension (R)_90	(EU)	436±20[c]	355±8[b]	286±11[a]	328±1[ab]
	R/E ration_90		2.2±0.1[a]	2.1±0.1[a]	2.0±0.1[a]	3.1±0.1[b]
	Energy_90	(cm^2)	200±9[c]	113±1[b]	66±1[a]	51±1[a]
	Extensibility (E) _135	(mm)	199±4[d]	172±1[c]	141±6[b]	94±3[a]
	Resistance to extension (R) _135	(EU)	434±4[c]	332±4[b]	296±9[a]	334±13[b]
	R/E ratio_135		2.2±0.1[a]	1.9±0.1[a]	2.1±0.1[a]	3.5±0.1[b]
	Energy_135	(cm^2)	202±12[c]	108±3[b]	67±1[a]	46±3[a]
EXTENSIONAL PROPERTIES (Alveograph Test)	Tenacity (P)	(mmH_2O)	108±6[a]	114±5[a]	141±4[b]	n.d.
	Extensibility (L)	(mm)	83±9[c]	49±5[b]	28±4[a]	n.d.
	P/L		1.3±0.2[a]	2.4±0.3[b]	5±1[c]	n.d.
	Force (W)	(10^{-4} J)	347±18[c]	224±15[b]	174±21[a]	n.d.
LEAVENING PROPERTIES (Rheofermentograph Test)	Maximum dough height	(mm)	52.6±2.1[c]	-	40.9±1.0[b]	30.4±0.2[a]
	Time of maximum dough development	(min)	160±25[b]	-	138±9[a]	140±1[a]
	Dough height at 360 min	(mm)	43.6±0.6[c]	-	32.8±1.1[b]	27.0±0.1[a]
	Total CO_2	(mL)	1797±22[b]	-	1602±86[ab]	1298±21[a]
	Retained CO_2	(mL)	1402±11[b]	-	1315±63[a]	1126±11[a]
	Released CO_2	(mL)	395±11[c]	-	286±23[b]	172±8[a]
	CO_2 retention coefficient	(%)	78.1±0.4[a]	-	82.2±0.5[b]	86.8±0.5[c]

Different letters in the same row correspond to significant differences (one-way ANOVA, Tukey test HSD, p ≤ 0.05). EU: Extensograph Units; FU: Farinograph Units; GPU: GlutoPeak Unit; n.d. not determined.
